# The analysis of the geographical distribution of emergency departments’ frequent users: a tool to prioritize public health policies?

**DOI:** 10.1186/s12889-021-11682-z

**Published:** 2021-09-16

**Authors:** Romain Hellmann, Anne-Laure Feral-Pierssens, Alain Michault, Enrique Casalino, Agnès Ricard-Hibon, Frederic Adnet, Dominique Brun-Ney, Donia Bouzid, Axelle Menu, Mathias Wargon

**Affiliations:** 1 Health Regional Agency of Ile de France, Paris, France; 2 Emergency Department, Bichat hospital, Assistance Publique-Hôpitaux de Paris, Paris, France; 3 SAMU 93 - Emergency Department, Avicenne hospital, Assistance Publique-Hôpitaux de Paris, Bobigny, France; 4 University Sorbonne Paris Nord, Health Education and Practices Laboratory (LEPS EA3412), Bobigny, France; 5 CIUSSS Nord de l’île de Montréal, Québec Montréal, Canada; 6 Conservatoire National des Arts et Metiers, Paris, France; 7 Paris University, INSERM, IAME, F-75006, Paris, France; 8 Emergency Department, Centre hospitalier René Dubos, Pontoise, France; 9 Direction de l’organisation médicale et des relations avec l’université, Assistance Publique-Hôpitaux de Paris, Paris, France; 10 Emergency Department, Centre Hospitalier de Saint-Denis, Saint-Denis, France; 11 Observatoire Regional des Soins Non Programmés - Ile-de-France, Saint-Denis, France

**Keywords:** Healthcare use, Frequent users, Access to care, Health geography, Emergency department

## Abstract

**Background:**

The individual factors associated to Frequent Users (FUs) in Emergency Departments are well known. However, the characteristics of their geographical distribution and how territorial specificities are associated and intertwined with ED use are limited. Investigating healthcare use and territorial factors would help targeting local health policies. We aim at describing the geographical distribution of ED’s FUs within the Paris region.

**Methods:**

We performed a retrospective analysis of all ED visits in the Paris region in 2015. Data were collected from the universal health insurance’s claims database. Frequent Users (FUs) were defined as having visited ≥3 times any ED of the region over the period. We assessed the FUs rate in each geographical unit (GU) and assessed correlations between FUs rate and socio-demographics and economic characteristics of GUs. We also performed a multidimensional analysis and a principal component analysis to identify a typology of territories to describe and target the FUs phenomenon.

**Results:**

FUs accounted for 278,687 (11.7%) of the 2,382,802 patients who visited the ED, living in 232 GUs. In the region, median FUs rate in each GU was 11.0% [interquartile range: 9.5–12.5]. High FUs rate was correlated to the territorial markers of social deprivation. Three different categories of GU were identified with different profiles of healthcare providers densities.

**Conclusion:**

FUs rate varies between territories and is correlated to territorial markers of social deprivation. Targeted public policies should focus on disadvantaged territories.

## Background

As most developed countries, France faces a regular and sharp increase of emergency departments (EDs) visits. In 2016, the 723 French EDs received nearly 21.2 million visits (+ 15% in 4 years) which account for €3.1 billion visits, mainly handled by universal health insurance system [1]. Improving health care access to unscheduled care facilities has become a national priority [2]. For several years, public policies have focus on the extension of general practitioner’s (GPs) visiting hours (on week nights and week-ends) or the development of alternatives for unplanned emergency care while the number of EDs has decreased over the same period [3]. Few interventions have targeted specific populations by promoting alternatives for unplanned care and health care pathways different than those involving ED visits. For example, frequent emergency users (FUs) have been described and successfully targeted in other health care systems [4–9]. FUs have not been investigated in France yet.

The Ile-de-France Health Regional Agency, the administrative institution that runs public health policies and the regulation of health services in the Paris metropolitan area (with the highest density of population: 12.1 million inhabitants), decided to set up a specific public health strategy aiming at improving adequate health care access for FUs. Identifying the territories associated to higher FUs rates and thus, the most eligible to these specific interventions is an important first step. The main aim of this work is to describe and characterize FUs in this specific area. The secondary objective is to identify the geographical distribution of FUs at residential territory level.

## Methods

### Study setting and design

We performed a retrospective study based on the Universal Health Insurance’s claims database (Système national des données de santé-SNDS) [10].

The Universal Health Insurance (UHI) fund covers more than 90% of the French population. The French health care system consists of primary care (mainly private practice) and of hospital care (mainly public sector) and has a cost sharing policy. The UHI fund reimburses physician private practice on the basis of a national fee schedule with reimbursement rates ranging from 30 to 100% of the statutory tariff for each type of procedure. These tariffs are set by national agreements among physicians’ trade unions and the UHI fund. For emergency department visits, tariffs and out-of-pocket payments are the same in all types of healthcare facilities. Personal health expenditure is mostly financed by UHI fund (79%). The remaining is financed by public or private complementary health insurance (14%) and out-of-pocket payments (7%) [11]. The complementary universal health coverage (CMU-C) is a public complementary health insurance for the poorest part of the French population (8%). Foreigners without any legal status can benefit from a specific public health insurance plan (*Aide Médical d’Etat* - AME).

This SNDS administrative database includes all outpatient visits (in private practice or healthcare facilities), inpatient admissions, medical procedures, medications, imaging, that are partially or fully covered under the universal health insurance fund. It contains also patient’s characteristics such as age, sex and municipality of residence. Patients presenting with one of 30 specific chronic long-term conditions (among which diabetes, coronary artery disease, heart or lung failure, psychiatric conditions, cancer, severe stroke, HIV, tuberculosis) are supported by a specific comprehensive coverage system for all related care. Each patient affected by one of these diseases needs physician certificates and administrative approval to benefit from this system. There are no complementary or out-of-pocket fees associated to these specific conditions. Administrative data concerning these conditions are also reported (*Affection Longue Durée* – ALD).

We included all patients who visited at least once any ED of the Ile-de-France region (IdF) between January 1st to December 31st, 2015. We excluded all visits for obstetric emergencies and childbirths. The IdF area is a region composed of eight *départements*, including the city of Paris. It has 87 general and 35 pediatric EDs.

The clinical severity of each ED visit was assessed according to the following classification:

- need of a medical consultation only (level 1);
- need of a technical procedure (biology test, imaging exam) (level 2)
- need of a specialist opinion but without hospital admission (level 3);
- resulting in hospital admission (level 4).

### Definitions

There is no consensus in the literature on the definition of FU which ranges from ≥2 to ≥20 visits per year [4]. For this study, the definition of FU was based on the natural break in the distribution of ED visits in the studied population which allowed us to identify a cutoff of ≥3 visits per patient per year in any ED. This definition is also the most common one reported in the literature. Three subtypes of FUs were defined: low-FUs: [3–6] visits/year; high FUs: [7–19] visits/year; and very high FUs ≥ 20 visits/year [8].

### Geographical units

We assessed a territorial division of the IdF region which is divided in 8 administrative *departments*. We identified 232 geographical units (GUs). GUs were defined according to the National Institute of Statistics and Economic Studies (INSEE) methodology: For the *department* of Paris, GUs correspond to districts (*n* = 20); For the three *departments* closest to Paris, GUs correspond to municipalities (*n* = 123); For the four peripheral and larger *departments*, GUs correspond to either municipalities (*n* = 10) or townships (*n* = 79) according to the size of the population. INSEE has developed this geographical subdivision to make the statistical data of the smallest municipalities more reliable. To characterize each GU, we used thirty descriptive variables of the demographic and socio-economic status of its residential population: age distribution, income level, jobs typology, education level, family composition, densities of healthcare professionals and the Human Development Index-2 (HDI2). The HDI-2 is an index which takes into account the three dimensions of the Human Development Index (health, education, standard of living) adapted to the French situation and available for each GU [12, 13].

### Endpoints

The first objective of this study was to describe FUs in the specific IdF area. Our first endpoint is the FUs rate in the IdF region and at residential territory level. The secondary objectives of this study were to identify and characterize in terms of socio-demographics factors the territories the most associated to high FUs rates. Our secondary endpoint was to assess correlations between socio-demographics and economic characteristics of the geographic units and the FUs rate and to identify a typology of territories.

### Statistical analysis

We defined the ED visiting rate by assessing the total number of ED visits per 1000 residents for each GU. All ED visits were recorded and accounted for even when the visit took place in a different GU than the patient’s residential GU. Then, the FU’s rate was assessed based on the number of FUs residents in a GU reported to the population of all GU residents that had visited at least once any ED in the IdF region.

We assessed correlations with the FU rate of 151 available demographic and socio-economic descriptive variables of all geographical units: age distribution, income level, jobs typology, education level, family composition, densities of healthcare professionals and the HDI-2. Among these, we identified 30 descriptive variables that were correlated to FU’s rate. Fifteen active interest variables were favored for principal component analysis (PCA) [14]. We moved from 15 variables to four main components while keeping more than 92% of the information. We performed a second step analysis by using a hierarchical ascending classification from these four main components. This method allowed the identification and differentiation of three different classes of homogeneous GUs with similar characteristics.

Data management and statistical analyses were performed with Excel® software, Microsoft Office Professional Plus 2010 for Windows® version; SAS Enterprise Guide®, version 7.13 of SAS System for Windows®; and IBM SPSS®, Statistics 20 version for Windows®.

### Authorization

This study is based on public data extracted from the SNDS. Access to the data was granted to the authors as authorized personnel of the IdF Health Regional Agency. Conditions of access to the data are described in the French Decree No 2016–1871 of December 26th, 2016.

## Results

### FU’s rate

In 2015, 2,382,802 million patients (19.8% of the IdF population) visited at least once an ED of the IdF region, accounting for 3,718,209 million visits. Among those visits, 44.6% of them were due to 1.6 million patients who visited the ED only once. FUs accounted for 278,687 patients, representing 11.7% of all ED patients and 30.8% of all ED visits (Table 1). Low FUs, High FUs, and Very High FUs accounted respectively for 23, 7.2 and 0.6% of all ED visits (10.8, 0.9 0.02% of all ED patients). FUs represent 2.3% of the IdF population.

**Table 1:** Demographic and medical characteristics of Emergency Department users and their visits in the Ile de France region in 2015 (IdF area)

Variables	IdF area	%	NFUs & FUs	%	FUs	%	NFUs	%
**Population, n**	12,055,277	100	2,382,802	100	278,687	100	2,104,115	100
**Men, n**	5,822,699	48.3	1,146,128	48.1	121,904	43.7	1,024,572	48.7
**Median age, years**	36		31		30		32	
**Chronic long-term condition**^a^**, n**								
Diabetes	454,210	3.8	90,115	3.8	12,670	4.5	77,445	3.7
Psychiatric disorder	243,626	2.0	60,867	2.5	12,341	4.4	48,526	2.3
Cancer	371,109	3.1	76,872	3.2	11,085	3.9	65,787	3.1
Chronic heart feature	152,938	1.3	42,056	1.8	6850	2.4	35,206	1.7
Coronary artery disease	178,234	1.5	42,588	1.8	6137	2.2	36,451	1.7
Chronic respiratory failure	99,224	0.8	27,431	1.1	5232	1.9	22,199	1.0
Severe hypertension	154,481	1.3	33,729	1.4	4946	1.8	28,783	1.4
Dementia	59,091	0.5	19,929	0.8	3335	1.2	16,594	1.4
			**NFUs & FUs Visits**	**%**	**FUs Visits**	**%**	**NFUs Visits**	**%**
**Visits, n**			3,718,209	100	1,144,837	100	2,573,372	100
**Degree of severity, n**								
Level 1 – medical consultation only			790,627	21.3	219,210	19.1	571,417	22.2
Level 2 – need for technical procedure			1,518,493	40.8	540,393	47.2	978,100	38.0
Level 3 – need for specialist opinion			842,816	22.7	201,903	17.6	640,913	24.9
Level 4 – hospital admission			566,273	15.2	183,331	16.0	382,942	14.9

The FUs represent 6.6% of ED patients aged [0–4] years old, 2.9% of the [25–34] y.o, and 5.7% of the patients aged ≥85 y.o. Nineteen per cent of FUs benefit from CMU-c vs 11% of non-FUs (NFU). Moreover, 0.5% of FUs benefit from AME vs 0.3% for NFUs.

Sixteen per cent of FU visits resulted in hospital admission vs. 14.9% for NFUs visits. Complementary procedures were necessary for 47.2% of FUs visits (38% for NFUs). Among the ED visits that did not result in hospital admission, 22.2% consisted in a simple physician consultation, 17.5% needed at least one biology test, 16.5% at least one imaging exam, and 4% in at least one biology test and imaging exams. Characteristics of ED visits for FUs and NFUs are listed in Table 1.

FUs: Frequent users: ≥ 3 visits/year. NFUs: Not Frequent Users: 1 to 2 visits/year. ^a^ Patients benefiting from the Comprehensive health coverage system for chronic health conditions (ALDs).

### Primary care use

Among all FUs 28.1% of them visited a general practitioner (GP) within 48 h before their ED visit, compared to 51.6% of NFUs (*p* <  0.000 Cramer’s V: 0.240). Furthermore, 84.7% of FUs had visited at least once a GP over the study period. On average, FUs visited a generalist twice a year. In addition, 73.7% of all FUs visited a specialist physician at least once over the study period (with an average of three visits per year).

### FU’s rate and territorial characteristics

At GUs level, we report a median annual rate of ED visits of 189.2 ‰ residents with an interquartile range [IQR] from 148.5 to 229.9. When analyzing the 232 GUs of the IdF region, the FU rate varied from 3.7 to 17.4% with a median of 11.0% [IQR: 9.5–12.5]. Correlations between the characteristics of the geographic units and the FU rates are reported in Table 2. The density of GPs was not correlated with FUs rate whereas the density of pediatricians was negatively correlated with FUs rate.

**Table 2:** Correlations between the rate of frequent users in their geographical units and their socioeconomic markers (Ile-de-France region, 2015)

Variables	Rules	Correlation coefficients	***P***-value
**Population, Age**			
< 3 years (%)	Active variable	+ 0.54	< 0.001
[3–15 [years (%)	Active variable	+ 0.31	< 0.001
[15–35 [years (%)	Illustrative variable	+ 0.26	< 0.001
[35–75 [years (%)	Active variable	−0.50	< 0.001
≥ 75 ans years (%)	Active variable	−0.38	< 0.001
**Education level**			
College degree (%)	Active variable	−0.55	< 0.001
Professional degree (%)	Active variable	+ 0.26	< 0.001
Bachelor degree (%)	Illustrative variable	+ 0.07	0.284
High school degree (%)	Active variable	+ 0.69	< 0.001
**Occupational status**			
Farmers (%)	Illustrative variable	+ 0.07	0.263
Employees (%)	Active variable	+ 0.58	< 0.001
Manual laborers (%)	Active variable	+ 0.61	< 0.001
Traders, Artisans (%)	Illustrative variable	−0.27	< 0.001
Middle managers (%)	Illustrative variable	−0.28	
Managers (%)	Active variable	−0.50	< 0.001
Pensioners (%)	Active variable	+ 0.39	< 0.001
Unemployed (%)	Active variable	+ 0.46	< 0.001
**Tax and income level (mean)**			
Taxable households (%)	Active variable	−0,74	< 0.001
Salaries and wages (€)	Active variable	−0,64	< 0.001
Pension and retirement (€)	Active variable	−0,69	< 0.001
Direct taxes (€]	Illustrative variable	−0,46	< 0.001
**Developpment Index**			
HDI-2	Illustrative variable	−0,67	< 0.001
**Family composition**			
Single parents families (%)	Illustrative variable	+ 0,58	< 0.001
Families without children (%)	Illustrative variable	−0.46	< 0.001
Families with one child (%)	Illustrative variable	+ 0,27	< 0.001
Families with two children (%)	Illustrative variable	−0,15	< 0.023
Families with three children (%)	Illustrative variable	+ 0,51	< 0.001
Families with at least three children (%)	Illustrative variable	+ 0,55	< 0.001
**Health care providers**			
General physicians density^b^	Illustrative variable	−0.03	0.660
Pediatricians density^c^	Illustrative variable	−0.24	< 0.000

^a^HDI-2: Human Developpment Index 2. ^b^Density > 100 general physicians per 100,000 residents. ^c^Density > 40 pediatricians per 100,000 residents under the age of 18

### Classes of geographical units

**Fig. 1:**
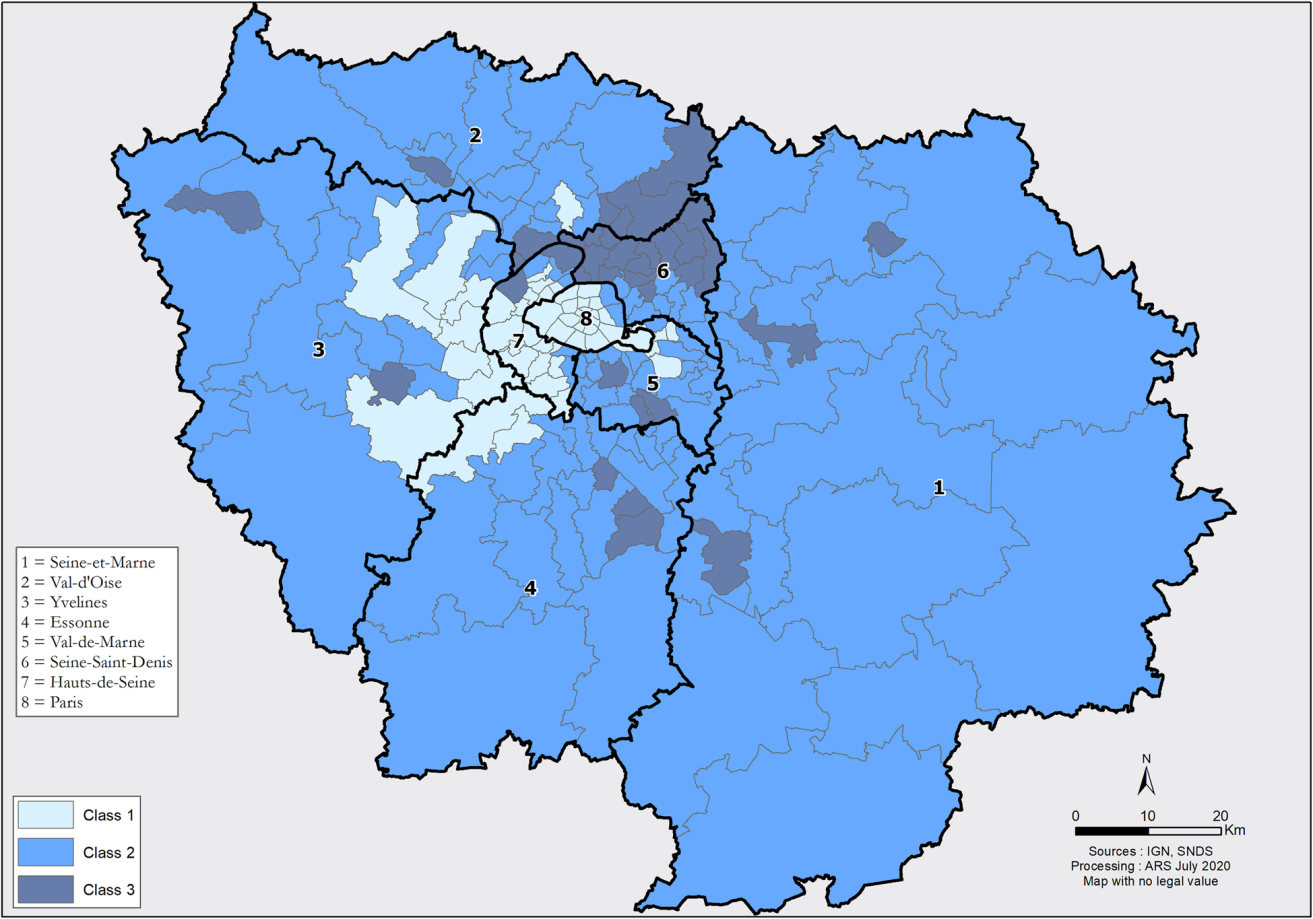
Geographical distribution of the three classes of territories among the 8 *departments* of the Ile-de-France area, 2015 (own source). Class 1: Low Frequent Users rates; Class 2: Average Frequent Users rates; Class 3: Higher Frequent Users rates

**Fig. 2:**
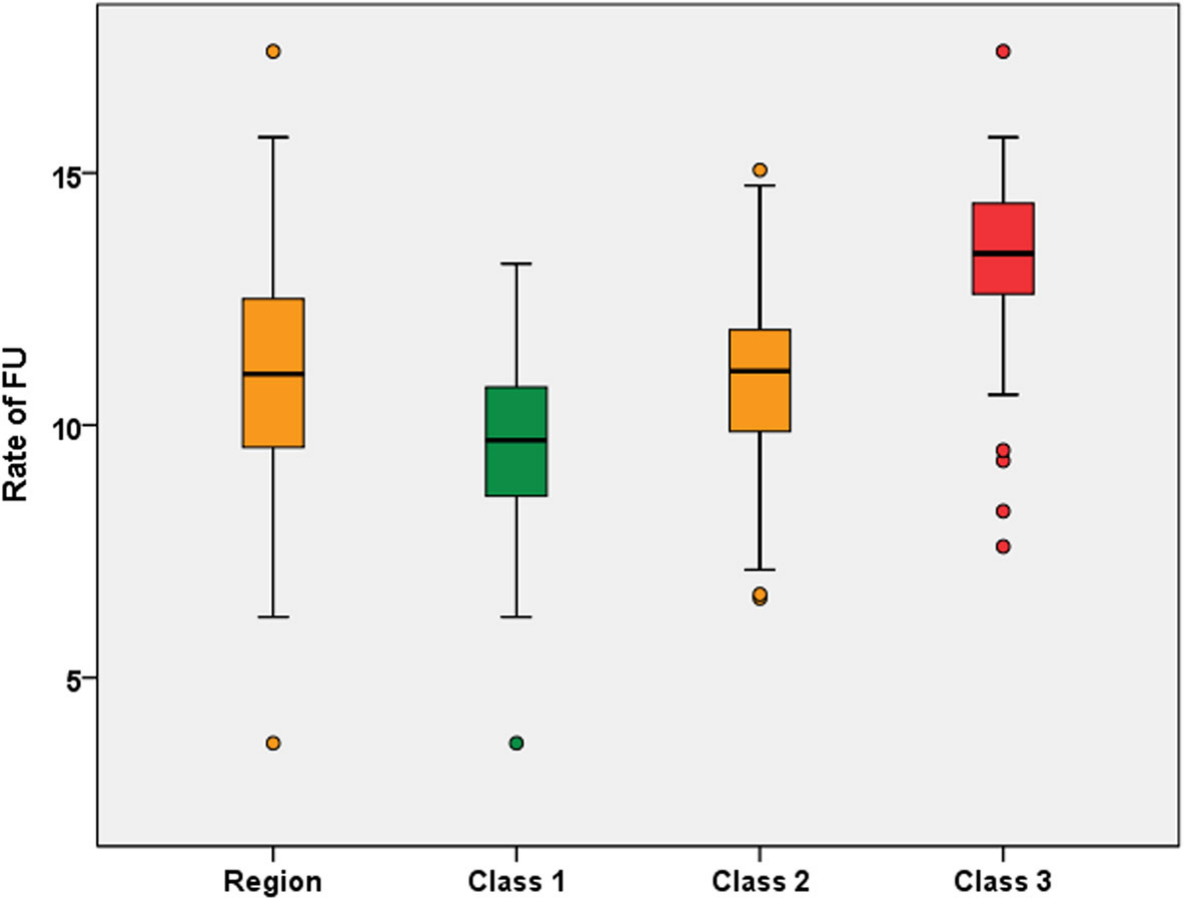
Rate of frequent users in each class of geographical units (IdF area, 2015). Caption: For the class 1 geographical units, the median rate of FUs is 9.7% with an interquartile range from 8.6 to 10.8%.

**Fig. 3:**
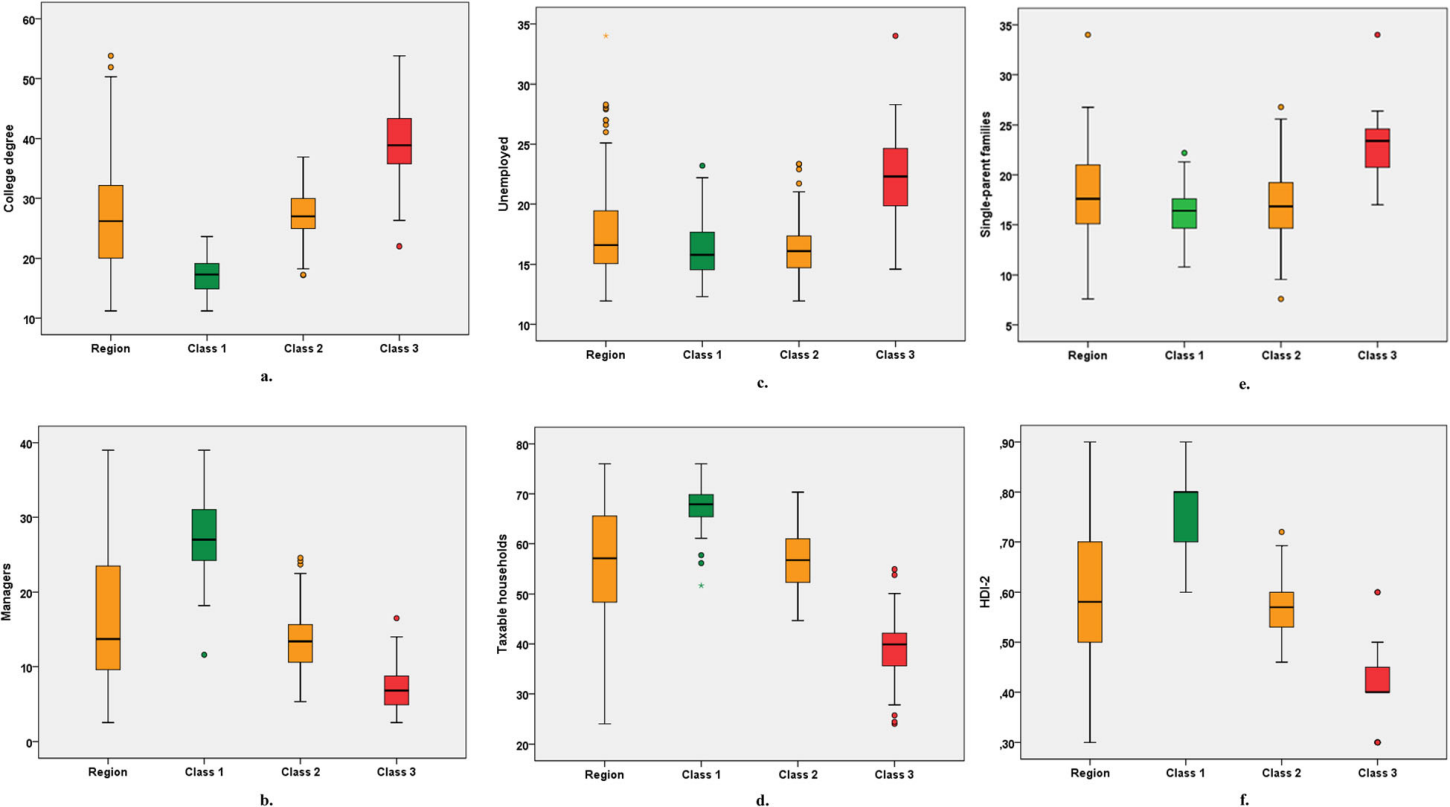
**A**. Rate of individuals with college degree in each class of geographical units. Caption: For class 1 geographical units, the median rate of individuals with a college degree is 17.3% with an interquartile range from 14.8 to 19.2%. **B**. Rate of managers in each class of geographical units. **C**. Rate of unemployed in each class of geographical units.  **D**. Rate of taxable households in each class of geographical units.  **E**. Rate of single-parent families in each class of geographical units. **F**. Median and interquartile range of the Human Development Index-2 in each class of geographical units. (IdF area, 2015)

**Fig. 4:**
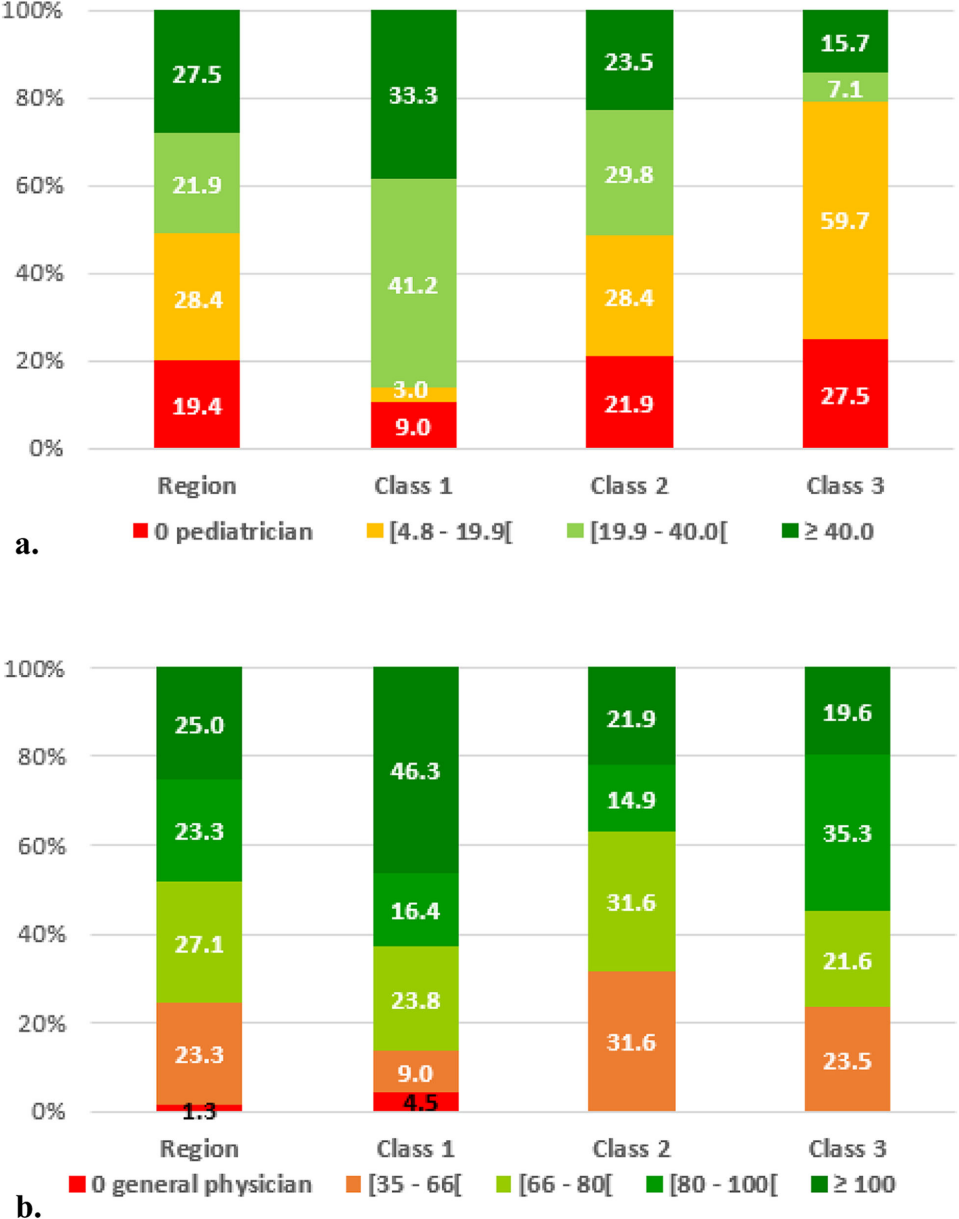
**A.** Rate of geographical units in each class depending on their pediatrician density (IdF area, 2015). **B**. Rate of geographical units in each class depending on their general physician density (IdF area, 2015)

The hierarchical ascending classification resulted in three classes of GUs in the IdF area. Their geographical partition is illustrated in Fig. 1. Two of the 3 classes identified present major differences in terms of socio-economic indicators (classes 1 and 3). The profile of class 2 indicators is close to the regional level. Inter-class inertia (9.0) covers 64.8% of the total inertia (13.9). The class 1 is characterized by wealthy socio-economic conditions, included 67 GUs (32.6% of IdF population), while class 3 included 51 GUs and social deprivation markers (20.5% of IdF population). Within class 3, the IQR of FU rates ranged from 12.6 to 14.4% (median 13.4%) vs 8.6 to 10.8% (median 9.7%) for Class 1 (Fig. 2, Table 3). When comparing class 1 and 3, there were higher socio-professional profiles in class 1 with more individuals graduating from higher educational degrees and 3.8 times more executives than in class 3. Conversely, GUs of class 3 reported more inactive individuals and 1.4 times more single-parent families. All results are presented in Figs. 3a-f and in Table 3. Regarding health care providers, class 1 included territories with a higher pediatrician’s density (3.8 times more territories with pediatric densities over 40 pediatricians per 100,000 people under 18) and GPs density (2.4 times more than 100 GPs per 100,000 inhabitants) (Table 4 and Fig. 4a and b). In class 1, the proportion of FUs over 75 years-old was higher than in the other two classes (16.2% vs. 6.8%). In contrast, class 3 had a higher FU rate for ≤3 years-old.

**Table 3:** Socio-economic characteristics among the residential population for each class of geographical units of the Ile-de-France region, 2015

Characteristics	Minimum*	Q1	Median	Q3	Maximum*
**Frequent Users, %**					
**Region**	6.2	9.5	11.0	12.5	15.7
**Class 1**	6.2	8.6	9.7	10.8	13.2
**Class 2**	7.1	9.9	11.1	11.9	14.7
**Class 3**	10.6	12.6	13.4	14.4	15.7
**College degree, %**					
**Region**	11.2	20.0	26.2	32.2	50.3
**Class 1**	11.2	14.8	17.3	19.2	23.6
**Class 2**	17.3	24.9	27.0	30.0	36.9
**Class 3**	26.3	35.5	38.9	43.9	53.8
**Managers, %**					
**Region**	2.5	9.6	13.7	23.5	39.0
**Class 1**	18.2	24.0	27.0	31.1	39.0
**Class 2**	5.3	10.5	13.4	15.7	22.5
**Class 3**	2.5	4.9	6.8	8.9	14.0
**Unemployed, %**					
**Region**	11.9	15.0	16.6	19.4	25.1
**Class 1**	12.3	14.5	15.8	17.7	22.2
**Class 2**	11.9	14.7	16.1	17.4	21.0
**Class 3**	14.6	19.6	22.3	24.7	28.3
**Taxable households, %**					
**Region**	24.0	48.4	57.1	65.6	76.0
**Class 1**	61.1	65.4	67.9	70.0	76.0
**Class 2**	44.7	52.2	56.7	61.1	70.3
**Class 3**	27.8	35.6	39.9	42.3	50.1
**Single-parent families, %**					
**Region**	7.6	15.1	17.6	21.0	26.8
**Class 1**	10.8	14.6	16.4	17.6	21.3
**Class 2**	9.5	14.6	16.8	19.2	25.6
**Class 3**	17.0	20.6	23.4	24.8	26.4
**HDI-2**					
**Region**	0.30	0.50	0.58	0.70	0.90
**Class 1**	0.60	0.70	0.80	0.80	0.90
**Class 2**	0.46	0.53	0.57	0.60	0.69
**Class 3**	0.40	0.40	0.40	0.50	0.50

* Other than outliers. Q1: First quartile. Q3: Third quartile. HDI-2: Human Development Index 2

**Table 4:** Socio-economic characteristics of the different classes of geographical units (IdF area, 2015)

Characteristics *	IdF area	Class 1	***P***-value*	Class 2	***P***-value*	Class 3	***P***-value*
**Geographical units (n)**	232	67		114		51	
**Population, n**	12,055,277	3,934,850		5,644,896		2,475,531	
**%**	100	32.7		46.8		20.5	
**FU rate, mean (%)**	11.0	9.6	< 0,001	10.9	NS	13.3	< 0,001
**Education, mean (%)**							
College Degree	38.3	57.2	< 0,001	34.0	< 0,001	23.3	< 0,001
**Occupational status, mean (%)**							
Managers	16.1	27.3	< 0,001	13.6	< 0,001	7.2	< 0,001
Employees	17.4	12.3	< 0,001	18.2	0.001	22.2	< 0,001
Manual Laborers	9.3	4.3	< 0,001	9.9	NS	14.6	< 0,001
Unemployed	19.9	16.3	< 0,001	16.2	< 0,001	22.5	< 0,001
**Tax and income level, mean**							
Taxable households (%)	55.9	67.5	< 0,001	56.8	NS	39.0	< 0,001
Total amount of net tax (€ per year)	5473	10,106	< 0,001	4054	< 0,001	2557	< 0,001
Amount of salaries and wages (€ per year)	34,717	46,982	< 0,001	32,348	< 0,001	23,899	< 0,001
Amount of pensions and retirements (€ per year)	26,920	33,525	< 0,001	25,985	NS	20,334	< 0,001
**Family composition, mean (%)**							
Single-parent families	18.1	16.0	< 0,001	17.2	NS	22.9	< 0,001
Families with three children or more	12.2	9.3	< 0,001	11.3	NS	18.2	< 0,001
**Development index, mean**							
HDI-2	0.59	0.76	< 0,001	0.57	NS	0.41	< 0,001
HDI-2 < 0.52 (%)	28.4	0.0	< 0,001	17.5	< 0,001	90.2	< 0,001
**Health care providers density (mean per 10**^**5**^**inhabitants)**							
> 100 General Physicians	25.0	46.3	< 0,001	14.9	< 0,001	19.6	NS
> 40 Pediatricians	24.1	59.7	< 0,001	7.0	< 0,001	15.7	NS

NS: Not significant (*P* > 0.05): The difference between the region mean and the class mean is not statistically significant. FU: Frequent users: ≥ 3 visits/year. HDI-2: Human Development Index 2.

*P-value comparing for each class and each indicator the mean of mean per GU to regional average

## Discussion

Considering our results, Paris area is not spared with the FUs phenomenon, since they represent one tenth of all ED patients. These results are consistent with those usually reported in the literature, varying from 4.5 to 9.9% depending on the definitions as well as according to the health systems studied [4, 15]. While the individual factors determining the use of emergency care are well documented, few studies have addressed the association between the socio-economic indicators of the geographical environment and the use of EDs [16, 17]. Recently, Lee and al. reported geographic variation in the demand for emergency care [18]. The overall rate of emergency care use was correlated to the deprived socio-economic characteristics of a Census tract. The case of the FUs was not addressed in this approach. Thus, our study report that the FUs phenomenon follows the same variation as socioeconomic characteristics and indicators of residential GU (low-income level, low level of employment or education, and family structure). The high rate of FUs could thus become an indicator of poverty in these geographical areas and highlights social inequalities in access to health. Working on these inequalities through joint policies with other partners (such as city, education, or transportation actors) seems essential and might help in reducing the FU rate and increase the population’s health status.

In multidimensional model, FUs rate was not correlated with GPs density however class 1 and 3 present different profiles of healthcare providers densities. The evidence on the association between increasing the supply of primary care and lower ED visits was not univocal in several studies [18–21]. In light of ours results, we can challenge the assertion of efficiency of FU’s visits reduction Programs mainly based on the increase of primary care physicians. Moreover, we reported that FUs have primary care use close to that of the general population and visit a general practitioner twice a year (versus 2.7 for the general population). Nevertheless, it seems that contrary to NFUs, the majority of FU patients do not see a GP before visiting the ED even though, according to the Organization for Economic Co-operation and Development (OECD), France is one of the countries with an easy daytime access to physicians [21].

Accessibility to the health care system does not depend solely on medical density, but involves other determinants such as affordability, acceptability, availability [22]. In their study, Giebel et al. reported that in socially deprived areas, socio-economic determinants are associated with lower access to primary care and worse health status. These markers were associated with higher ED visiting rate [17]. In France, a recent study reported that patients who have forgone health care for financial issues, have indeed an increased use of EDs and a decreased use of primary care physicians [23]. Nevertheless, including other determinants of health access, such as the distance between patient’s residence and the nearest primary care facility or the availability of at-home GP visits could help refine our results. To reduce ED visits, it is also likely that public policies are needed to improve accessibility by urban transit to primary care services and to provide better housing and working conditions [17].

There are several typologies of FU in the literature [4, 24]. In our study, the prevalence of different chronic diseases reported for FUs are higher than those observed in the general population. This trend is consistent with literature [25]. People with chronic illnesses and psychiatric disorders are more likely to visit EDs; this also holds true for people with low socio-economic status [16]. These sub-populations of FU may be different targets for action plans or specific approaches such as case management, improved coordination of care or health education [6, 26–28]. These individual plans are often based on the principle of secondary prevention because they are triggered after an ED visit through the detection of a FU’s pattern. Primary prevention for FUs phenomenon is not much discussed in the literature. Our study shows a correlation between FUs environmental factors (characteristics of the geographical unit) and the FU rate. Thus, it seems possible for institution or health care providers to identify territories where residents are at higher risk of developing FUs pattern and to suggest primary prevention actions.

Geographical targeting of the FU phenomenon was an important aim of our study. Geolocalization of health issues makes it possible to address them in a multidimensional model [29]. It provides a better understanding of the links between human environmental factors (particularly socio-economic) and various public health issues [30]. This approach corresponds to the new orientations of health policies in France. Transforming our health system requires a better targeted, more relevant and more efficient response by defining priority areas for actions [31]. Our study has highlighted a concentrated geographical distribution of territories and municipalities that are the most affected by the FU phenomenon. This is a fundamental first step before adapting and embracing a territorial approach of public policies. It should also help mobilizing decision makers of various public services (education, health insurance, elected officials of the communes...) and press them to act. A tiled distribution of GU classes would jeopardize this kind of territorial approach. Indeed, the fragmentation of the IdF territory would dilute and scatter the means for actions even preventing to deploy.

However, our study presents some limitations. We defined two classes of emergency users based on the number of visits generated during 2015, frequent and non-frequent users. There are several definitions of multiple emergency users in the international literature [7]. Having no reference on the quantification of the FU phenomenon in France and following ED visits distribution for our population, the cutoff chosen for this study was 3 ED visit within the year under study. It is undoubtedly necessary to carry on more targeted studies with different cut offs to better specify the FU phenomenon in our region. Other limitations are related to the structuring of the databases that is only a reflection of the construction of the French health care system. The database used is issued from the universal health insurance claims database [11, 28]. While this database allows for almost complete exhaustivity on ED visits, some characteristics of the studied population could not be extracted (for example: the rate of ED visits for foreigners without legal status and without medical insurance). In addition, since the French universal health insurance provides only few medical and clinical information, it prevented us from giving a full medical profile of the FUs population.

## Conclusion

Like other countries, France and more precisely the Ile-de-France region (which comprises Paris) is not spared with Frequent Users of Emergency Departments. Frequent users represent 11.7% of all ED patients and 30.8% of all ED visits. Low socio-economic indicators among the IdF residential population is associated to higher rate of FUs. The high density of pediatricians is associated to lower rates of FUs whereas GPs density is not associated to lower rates of FUs. Analyzing the geographical distribution of FUs rate, we identify and describe three different classes of territories with different socio-economic profiles and different profiles of healthcare providers densities. Social deprivation markers at a geographical unit level are also associated to higher rates of FUs. Efforts to reduce this phenomenon may not only be carried out by health actors (public institutions, healthcare providers), but must include all public territorial actors such as education, transportation, elected officials. Transforming our health system requires a better targeted and a more efficient response by defining uphill priority areas for actions. These targeted public policies should focus on disadvantaged territories.

## Data Availability

The datasets generated and analyzed during the current study are not publicly available due to legal restrictions. The national legislation in France protects personal data and materials. Therefore, before any data transfer a legal authorization has to be obtained from CNIL, the French data protection authority.

## Abbreviations

ALD: Affection Longue Durée
AME: Aide Médicale d’Etat
CMU-c: Complementary Universal health coverage (Couverture Maladie Universelle Complémentaire)
ED: Emergency Department
FU: Frequent Users
GP: General Physician
GU: Geographical Unit
HDI: Human Developpment Index
IdF: Ile-de-France region
INSEE: National Institute of Statistics and Economic Studies
IQR: InterQuartile Range
NFU: Non Frequent Users
OECD: Organization for Economic Co-operation and Development
PCA: Principal Component Analysis
SNDS: Système National des Données de Santé
